# Prediction of Postoperative Ileus in Patients With Colorectal Cancer by Preoperative Gut Microbiota

**DOI:** 10.3389/fonc.2020.526009

**Published:** 2020-11-25

**Authors:** Ye Jin, Rui Geng, Yang Liu, Lujia Liu, Xiangren Jin, Fuya Zhao, Jing Feng, Yunwei Wei

**Affiliations:** ^1^Department of Oncological and Laparoscopic Surgery, The First Affiliated Hospital of Harbin Medical University, Harbin, China; ^2^Department of Hepatic Surgery, The First Affiliated Hospital of Harbin Medical University, Harbin, China; ^3^Key Laboratory of Hepatosplenic Surgery, Ministry of Education, The First Affiliated Hospital of Harbin Medical University, Harbin, China; ^4^Department of Thyroid and Breast Surgery, The First Affiliated Hospital of University of Science and Technology of China, Hefei, China

**Keywords:** gut microbiota, ileus, colorectal cancer, postoperative ileus, *Faecalibacterium*

## Abstract

**Background:**

Ileus and postoperative ileus (POI) are common complications of colorectal cancer (CRC). However, little is known about the gut microbiota associated with ileus.

**Method:**

Differences in gut microbiota were evaluated by 16S rRNA gene sequencing. We characterized the gut microbiota in 85 CRC patients (cohort 1) and detected differences, and an independent cohort composed of 38 CRC patients (cohort 2) was used to evaluate the results.

**Results:**

The gut microbiota of CRC patients with and without ileus exhibited large differences in alpha- and beta-diversities and bacterial taxa. The Firmicutes-to-Bacteroidetes ratio and microbial dysbiosis index (MDI) showed greater dysbiosis among ileus patients than among those without ileus. According to the location of CRC, the difference in gut microbiota between patients with and without ileus was more obvious in those with distal CRC than in those with proximal CRC. Finally, *Faecalibacterium* was significantly reduced in the postoperative perioperative period in patients with ileus. Thus, we used *Faecalibacterium* as a biomarker for predicting perioperative or POI: the AUC value was 0.74 for perioperative ileus and 0.67 for POI that appeared at 6 months after hospital discharge. The predictive power was evaluated in Cohort 2, with an AUC value of 0.79.

**Conclusion:**

These findings regarding difference of gut microbiota in postoperative CRC patients may provide a theoretical basis for the use of microbiota as biomarkers for the prediction of POI.

## Introduction

Colorectal cancer (CRC) has the third highest incidence and second highest mortality rate among cancers (1). Approximately 10% of cases will be diagnosed in an emergency context because of ileus (2), a life-threatening situation that requires immediate surgery. CRC recurrence after surgical resection is the main cause of treatment failure, and overall recurrence rates are significantly higher after obstruction and subsequent emergency resection than after elective resection (3).

Emerging evidence suggests that gut microbial dysbiosis is an important environmental factor contributing to CRC (4–6). Indeed, researchers have put a lot of effort into defining the microbiota in colorectal carcinogenesis (7, 8). Additionally, ileus with increased fecal retention is thought to promote overgrowth of the microbiota (9), though this is largely based on bacterial culture studies. However, with the development of next-generation sequencing technology, recent studies have found a significantly negative correlation between microbiota richness and colon transit rate (10). Furthermore, molecular-level data demonstrate that the intestinal microbiota can regulate bowel motility (11). Nonetheless, how ileus affects community dynamics of the microbiota and whether microbiota alterations contribute to pathophysiological changes, remain unknown.

As a common complication after surgical resection of CRC (12), the incidence of postoperative ileus (POI) is reportedly 10.2% (13). POI is closely associated with poor survival, delayed postoperative recovery, and longer hospital stays and also leads to significant reduction in quality of life and substantial health care costs (14, 15). A recent clinical study showed that preoperative mechanical bowel preparation with oral antibiotics significantly reduced ileus after colorectal surgery (16). In other words, POI should be anticipated, and efforts to reduce its duration should begin preoperatively. Although Bragg et al., summarized the strategy for preventing POI (12), there are no specific biomarkers available to accurately predict its occurrence.

For earlier POI detection, we designed a clinical research of the 16S rRNA gene sequence using mucosa samples of CRC patients with and without ileus. We characterized the gut microbiota of 85 samples and detected differences. In addition, based on follow-up information, we researched the ability of gut microbiota from preoperative specimens to predict POI. Finally, an independent cohort composed of 38 CRC patients was used to evaluate the results. All these researches were expected to demonstrate that the gut microbiota can be a valuable tool for the identification of biomarkers to evaluate the POI in CRC postoperative patients.

## Materials and Methods

### Human Samples

In this prospective clinical trial, recruit participants and sample collection were carried out at the First Affiliated Hospital of Harbin Medical University from June 2016 to January 2019. Participants were older than 18 years but not older than 85 years, with a recent colonoscopy and without long-term antibiotic treatment. CRC was diagnosed by colonoscopic examination and histopathological review of biopsies. CRC patients with ileus were diagnosed if two or more of the following conditions were met: absence of flatus for more than 24 h; absence of stool for more than 72 h; abdominal distension; nausea or vomiting; and clinical and imaging examinations (including X-ray radiograph, sonography, CT, and colonoscopic examination) indicating ileus. POI was diagnosed if two or more of the following criteria were met on or after postoperative day 4: absence of flatus for over 24 h; absence of stool for over 72 h; abdominal distension; nausea or vomiting; cannot tolerate diet in the past 24 h; and clinical and imaging examinations (including X-ray radiograph, sonography, and CT) indicative of POI. Exclusion criteria were as follows: distant metastasis; permanent ostomy; history of autoimmune diseases, diabetes mellitus, or chronic diarrhea; inflammatory bowel disease; probiotics or antibiotics were used 3 months prior to collection; any history of cancer other than CRC; history of other abdominal surgery; any known disease that may affect the gut microbiota. Through screening, 85 eligible patients were selected and grouped into cohort 1. Of these, 34 patients have preoperative ileus and 51 do not have preoperative ileus. To verify the accuracy of the experimental results in queue 1, we applied an independent queue (queue 2, 38 patients) to verify the accuracy of the results.

### Sampling, DNA Extraction, and PCR Amplification

Mucosal samples were collected from colorectal tumor and subsequently evaluated by hematoxylin and eosin (H&E) staining. Biopsy samples were snap-frozen in cryovials immediately after biopsy and stored at −80°C until DNA extraction. Microbial DNA was extracted from the mucosa using NucleoSpin^®^ Tissue Kit (Macherey-Nagel GmbH & Co., Düren, Germany) according to the manufacturer’s instructions. The hypervariable region (V3–V4) of the bacterial 16S rRNA gene was amplified using a thermocycler PCR system (GeneAmp 9700, ABI, Natick, MA, United States).

### 16S rRNA Gene Sequencing and Data Processing

Purified amplicons were pooled in equimolar concentrations and sequenced using the Illumina MiSeq platform (Illumina, San Diego, CA, United States) in PE300 mode according to the standard protocols provided by Majorbio Bio-Pharm Technology Co., Ltd (Shanghai, China). 16S rRNA gene sequencing data were processed using the Quantitative Insights Into Microbial Ecology platform (QIIME; V.1.9.1). Operational taxonomic units (OTUs) were selected according to a cutoff of 97% similarity, and the identified taxa were aligned using the Greengenes database (V.13.8).

### Bioinformatics Analysis

Raw counts of 3,206,494 *de novo* OTUs were compiled into 46 phyla, 100 classes, 213 orders, 398 families, 929 genera, and 4736 OTUs. Richness greater than 0.5% among OTU sequence reads was used in taxonomic signature analysis and Venn, involving 6 phyla, 10 classes, 13 orders, 19 families, 25 genera, and 36 OTUs.

Alpha diversity was assessed by Sobs, Shannon, and Simpson indices. Beta diversity was estimated by computing unweighted UniFrac and weighted UniFrac distances and visualized using principal coordinate analysis (PCoA); the results were plotted using the WGCNA, stats, and ggplot2 packages in R software (Version 2.15.3).

Permutational multivariate analysis of variance (PERMANOVA) of distance matrices, as implemented in the “vegan” package in R, was employed to identify whether case/control status explained variation in the observed microbial community composition.

The mucosa samples were classified into enterotypes according to whether they displayed a similar microbial composition at the genus level. Based on the relative abundance at the genus level, the Jensen–Shannon distance was calculated, partitioning around medoid clustering was conducted, the optimal clustering *K* value was calculated through the Calinski–Harabasz index, and PCoA was performed for visualization.

To evaluate the discriminatory ability of the prediction model, receiver operating characteristic (ROC) curves were constructed, and area under the curve (AUC) values were calculated. The abundance of *Faecalibacterium* was used as a biomarker, and POI patients and controls were set as 0 and 1. The R package (pROC) was utilized for drawing ROCs and obtaining AUC values.

### Statistical Methods

All statistical analyses were performed using SPSS 23.0 software and the R package. Microbiotal features differentiating the mucosa microbiota were characterized using the linear discriminant analysis effect size (LEfSe) method. Only bacterial taxa with average abundances >0.5% were analyzed. Multiple hypothesis tests were adjusted using the Benjamini and Hochberg FDR, and differences were considered significant when the results were below a threshold of 0.05, and *p*-values < 0.05 were considered significant.

## Results

### Study Population

We included 85 CRC patients in the cross-sectional study: 34 patients with preoperative ileus and 51 without preoperative ileus in Cohort 1. The postoperative patients were further divided into subgroups of 10 patients with perioperative ileus and 9 with POI (within 6 months after discharge). To assess the diagnostic potential of selected biomarkers, an independent cohort (validation cohort) composed of 38 CRC patients, including 6 patients with perioperative ileus, was recruited. No significant differences in age or sex between any of the groups and controls were observed. The location of CRC before surgery (proximal or distal) was determined by colonoscopy and imaging. The clinical characteristics and demographics between groups are shown in Table 1 and Supplementary Table S1.

**TABLE 1:** Demographic and clinical characteristics.

	**Cohort 1**			**Cohort 2**		
	**Ileus**	**No ileus**	***p***	**Ileus**	**No ileus**	***p***
*N*	34	51		15	23	
Male/Female	17/17	33/18	0.18	7/8	11/12	0.94
Age, years	65.2 ± 10.4	65.1 ± 8.6	0.79	66.0 ± 9.1	62.9 ± 9.1	0.36
**Tumor location**						
Proximal	8	13	0.84	5	6	0.63
Distal	26	38		10	17	
CEA, ng/ml	20.3 ± 36.2	60.1 ± 201.9	0.85	44.9 ± 72.9	89.6 ± 384.2	0.64
CA199, U/ml	40.3 ± 66.0	78.4 ± 204.6	0.81	111.1 ± 251.1	24.2 ± 46.8	0.14
AFP, ng/ml	2.7 ± 3.1	2.1 ± 1.2	0.80	1.5 ± 0.6	2.5 ± 1.8	0.06
Ferroprotein, μg/L	158.1 ± 166.6	173.8 ± 182.0	0.69	61.6 ± 81.4	207.4 ± 406.4	0.19
Diameter, cm	3.0 ± 1.7	3.3 ± 2.0	0.79	1.5 ± 0.8	1.3 ± 0.7	0.53

*The Wilcoxon rank-sum test was used to compare age, CEA, CA199, AFP, ferroprotein, and tumor diameter between the ileus and no-ileus groups. Fisher’s exact test was used to compare sex and tumor location distribution between the ileus and no-ileus groups. Values are expressed as the mean ± SD. AFP, α-fetoprotein; CA199, carbohydrate antigen 199; CEA, carcinoembryonic antigen.*

### Gut Microbiota Diversity and Composition in Ileus

To compare the difference of gut microbiota between CRC patients with or without ileus, we performed 16S rRNA sequencing from mucosa samples. After filtering, an average of 35,698 (range 26,388 to 60,796) reads per sample was obtained. Finally, each sample size was equal to 26,388.

**FIGURE 1:**
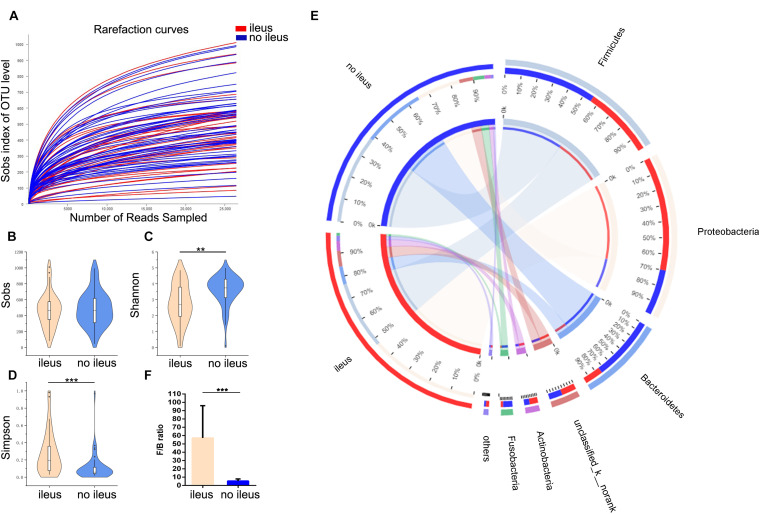
The shift of the gut microbiota composition in CRC patients with or without ileus. **(A)** The rarefaction curve reached a plateau, indicating that the sequencing depth was adequate. **(B–D)** The Sobs index, Shannon index, and Simpson index of the ileus and no-ileus groups were compared. **(E)** In the Circos plot, the small semicircle (left half circle) represents the species composition in the sample. The color of the outer ribbon represents the groups, the color of the inner ribbon represents the species, and the length of the ribbon represents the relative abundance of the species in the corresponding sample. The large semicircle (right half circle) indicates the distribution proportion of species in different samples at this taxonomic level. The color of the outer ribbon represents the species, the color of the inner ribbon represents the groups, and the length of the ribbon represents the relative abundance of the species in the corresponding sample. **(F)** The Firmicutes-to-Bacteroidetes ratio of the ileus and no ileus groups. Data are expressed as the means ± SD. The Wilcoxon rank-sum test, **p < 0.01, ***p < 0.001.

First, we studied the evenness and richness of the gut microbiota in CRC patients with or without ileus. Sequencing depth was measured by plotting rarefaction curves for richness (Figure 1A); most of the samples reached plateaus, indicating an adequate sequencing depth. Alpha diversity was evaluated at OTU level using Sobs, Shannon, and Simpson indices. The Wilcoxon rank-sum test revealed no significant difference in the Sobs index, which measures richness, between the groups with and without ileus (Figure 1B); however, the Shannon index, which measures evenness, was higher among those without ileus (Wilcoxon rank-sum test, *p* = 0.001, Figure 1C). Thus, we could not statistically demonstrate a difference in OTU-level richness between the two groups, even though the results indicated that overgrowth of certain bacteria altered the evenness of the gut microbiota in ileus patients. Consistently, the value for the Simpson index, which measures dominance in a community, was much higher in the ileus group than in the group without ileus (Wilcoxon rank-sum test, *p* = 0.0001, Figure 1D).

In addition, the gut microbiota structure was analyzed at the phylum level. The proportions of Firmicutes (43% vs. 57%), Bacteroidetes (23% vs. 77%), and Fusobacteria (20% vs. 80%) were lower, but those of Proteobacteria (71% vs. 29%) and Actinobacteria (61% vs. 39%) were higher in the group with ileus than in the group without ileus (Figure 1E); moreover, the Firmicutes-to-Bacteroidetes (F/B) ratio in the former was significantly higher than that in the latter (Wilcoxon rank-sum test, *p* = 0.0005, Figure 1F). The microbiota composition between the groups with and without ileus was also different at family and genus levels (Supplementary Figure S1).

**FIGURE 2:**
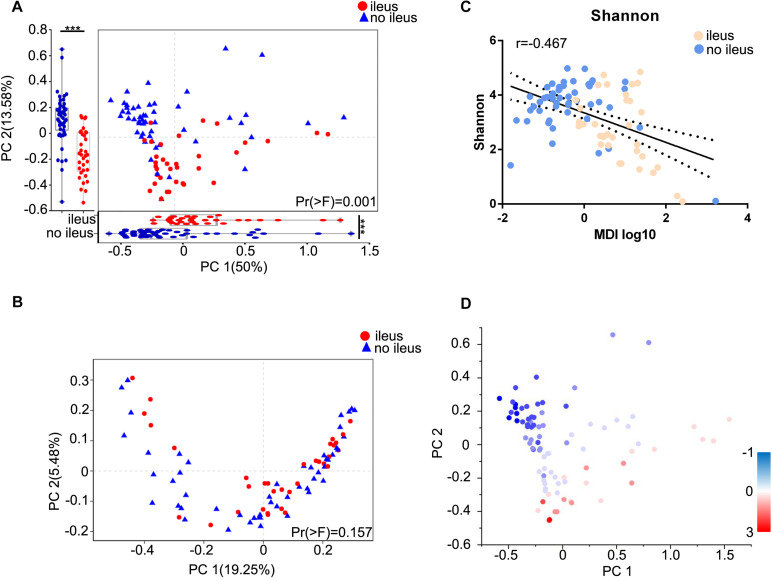
Diversity-associated dysbiosis of the gut microbiota in CRC patients with ileus or no ileus. Principal coordinate analysis of the **(A)** weighted UniFrac and **(B)** unweighted UniFrac distance between the ileus and no ileus groups. Projection axes were assessed individually by Wilcoxon rank-sum tests. The first two coordinates are plotted with the percentage of variability, as indicated on the axis. Each point represents a sample, and the colors represent different groups. **(C)** The relationship between the Shannon index and MDI for each sample. **(D)** The Shannon indices for each sample are represented by points on a weighted UniFrac distance PCoA, where a blue point represents a low value, a white point represents a mid-value, and a red point represents a high value. The Wilcoxon rank-sum test, ***p < 0.001. MDI, microbial dysbiosis index.

To assess the degree of similarity between the microbiota communities, both weighted and unweighted UniFrac phylogenetic distance matrices were used to calculated the beta diversity values and visualized in PCoA plots. The total diversity captured by the top two principal coordinates was 63.58 and 24.73% for the weighted and unweighted UniFrac distances, respectively. For weighted UniFrac, which considers the relative abundance of bacterial taxa, the gut microbiota of the CRC patients with ileus was clearly separated from that of the patients without ileus. Moreover, PC1 and PC2 were significantly different between the two groups. In contrast, there was no such segregation for unweighted UniFrac, which considers only the presence and absence of bacterial taxa [PERMANOVA test, Pr(>F) = 0.001 and Pr(>F) = 0.157 for weighted UniFrac and unweighted UniFrac distances, respectively, Figures 2A,B].

### Gut Microbial Dysbiosis and Bacterial Taxon Differences

We next combined the relevant taxa that characterized each group of patients and calculated the microbial dysbiosis index (MDI) (17) at the genus level. We found that the gut microbiota of ileus patients showed a higher MDI than that of the patients without ileus (Wilcoxon rank-sum test, *p* = 0.0011, Supplementary Figure S2D). The MDI exhibited an inverse correlation with alpha diversity (Spearman correlation analysis, *r* = −0.2946, *p* = 0.0062, Figure 2C) and resulted in a clear differentiation gradient among the samples with regard to beta diversity (Figure 2D). These results show a high degree of dysbiosis of ileus patients compared to those without ileus in the gut microbiota, consistent with the reduced bacterial diversity observed.

To find out the specific communities associated with CRC patients with ileus, the compositions of gut microbiota were compared in those with and without ileus using LEfSe analysis with an average abundance level >0.5%. A total of 31 discriminative features with an LDA > 3.5 were distinguished at the phylum (*n* = 4), family (*n* = 12), and genus (*n* = 15) levels (Supplementary Table S2). In addition, we applied the Mann–Whitney *U* test to compare the gut microbiota at different taxon levels by confining analyses, identifying 31 different abundant taxa at *p* < 0.05 and 30 different abundant taxa at *Q* < 0.05 (Supplementary Table S3). All of the identified taxa from the Mann–Whitney *U* test (*p* < 0.05) were consistent with the results of LEfSe analysis.

The abundance of Proteobacteria was high in the ileus group samples, whereas the abundances of Bacteroidetes, Firmicutes, and Fusobacterium were high in the no-ileus group samples. At the family level, the group with ileus exhibited enrichment of Enterobacteriaceae and Veillonellaceae, whereas Bacteroidaceae, Lachnospiraceae, Prevotellaceae, and Fusobacteriaceae, among others, were prevalent in the group without ileus. At the genus level, significantly greater abundances of *Escherichia–Shigella*, *Ralstonia*, and *Veillonella* were observed in the group with ileus than in the group without ileus; in contrast, levels of *Bacteroides*, *Prevotella 9*, *Fusobacterium*, and *Faecalibacterium*, among others, were decreased in the ileus group samples compared with those in the no-ileus group samples.

### Obvious Differences in Gut Microbiota in Distal CRC

**FIGURE 3:**
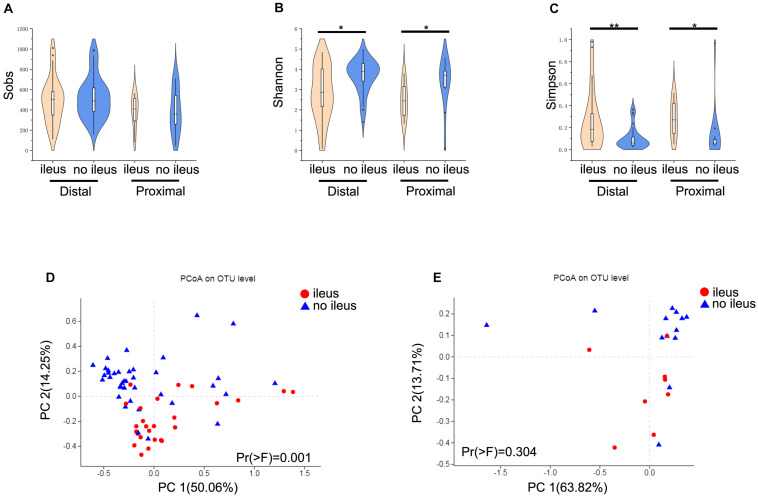
Different diversity of the gut microbiota in distal and proximal CRC patients. **(A)** The Sobs index, **(B)** the Shannon index, and **(C)** the Simpson index of the ileus and no-ileus groups of distal or proximal CRC on the OTU level. The Wilcoxon rank-sum test. Principal coordinate analysis of the weighted UniFrac distance between ileus and no-ileus group of **(D)** distal and **(E)** proximal CRC. *p < 0.05, **p < 0.01.

Next, we examined differences of gut microbiota with different locations (proximal and distal) of CRC and ileus. Interestingly, no significant differences in alpha and beta diversities between the proximal and distal groups were found (Wilcoxon rank-sum test, Supplementary Figure S2). Therefore, we compared gut microbiota differences based on CRC location in patients with and without ileus. Regarding alpha diversity, the results for proximal CRC + ileus/proximal CRC + no ileus comparisons and distal CRC + ileus/distal CRC + no ileus comparisons were consistent with the results mentioned above. Despite no significant difference in the Sobs index (Figure 3A), the Shannon index was clearly decreased, whereas the Simpson index increased (Wilcoxon rank-sum test, Figures 3B,C). The gut microbiota of the distal CRC + ileus group was clearly segregated from that of the distal CRC + no-ileus group based on the weighted UniFrac distance [PERMANOVA test, Pr(>F) = 0.001, Figure 3D]. Nevertheless, the weighted UniFrac distance showed no significant differences in the beta diversity of the gut microbiota for the proximal CRC + ileus group compared with the proximal CRC + no-ileus group [PERMANOVA test, Pr(>F) = 0.304, Figure 3E].

**FIGURE 4:**
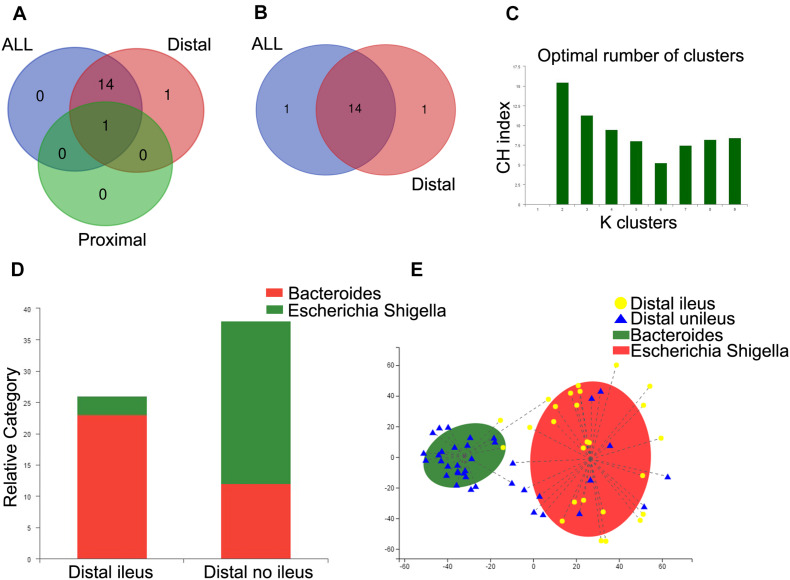
Enterotype analysis of distal CRC patients. **(A,B)** Venn diagram showing the common genera between groups. **(C)** When the values were fit to the partitioning around medoids, the optimal classification into two community types was indicated. **(D)** Distribution of the distal ileus and distal no-ileus samples in the community types. The areas of the columns are scaled to the sample size. Fisher’s exact test, *p* = 0.000002. **(E)** Plot of principal coordinate analysis of stool samples using partitioning around medoids.

We also applied the Mann–Whitney *U* test to compare the gut microbiota according to genera with abundance levels greater than 0.5% in these groups. Venn diagrams were employed to compare the different compositions of the gut microbiota among the ileus group vs. no-ileus group, proximal CRC + ileus group vs. proximal CRC + no-ileus group, and distal CRC + ileus group vs. distal CRC + no-ileus group at the genus level, at *p* < 0.05. This analysis revealed only one common bacterial genus: *Bacteroides*. The differential bacteria between the ileus vs. no-ileus groups and the distal CRC + ileus vs. distal CRC + no-ileus groups were almost entirely the same (Figure 4A). Moreover, according to *Q* value < 0.05, there was no significant difference in bacteria between the proximal CRC + ileus group and the proximal CRC + no-ileus group. In addition, the differences in bacteria between the ileus vs. no-ileus groups and distal CRC + ileus vs. distal CRC + no-ileus groups remained consistent, except for *Veillonella* among all patients and *Enterococcus* among distal CRC patients (Figure 4B). These results indicate that the difference in gut microbiota between patients with or without ileus is more obvious in those with distal CRC than in those with proximal CRC.

Furthermore, we analyzed differences in gut microbiota composition between the distal CRC + ileus and distal CRC + no-ileus groups through an enterotype classification according to genus abundance using partitioning around medoids. Two microbial community types were identified for distal CRC patients (Figure 4C). A large percentage of distal CRC + ileus patients (23/26) were observed to have the *Escherichia–Shigella* enterotype, with more distal CRC + no-ileus patients (26/38) having the *Bacteroides* enterotype (Figure 4D). PCoA revealed both distal CRC + ileus and distal CRC + no-ileus patients in each community type (Figure 4E).

**FIGURE 5:**
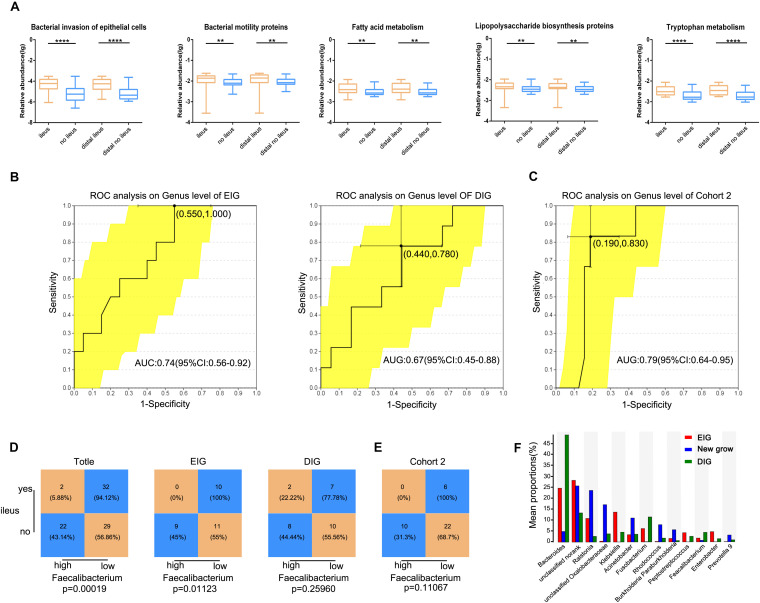
Gut microbiota prediction of postoperative ileus. **(A)** The five typically different KEGG pathways for the ileus, no-ileus, distal ileus, and distal no-ileus groups. The Wilcoxon rank-sum test. Receiving operating characteristic curve analysis was used to assess the predictive model performance of **(B)** EIG, DIG, and **(C)** Cohort 2. Statistical analysis was conducted based on the amount of *Faecalibacterium* and the ileus rate in **(D)** Cohort 2, EIG, DIG, and **(E)** Cohort 3 by the cutoff value of *Faecalibacterium* defined in Cohort 2, Chi-square test. **(F)** The abundance of the gut microbiota in the EIG, DIG, and new groups. **p < 0.01, ****p < 0.0001, EIG, ileus in the postoperative perioperative period; DIG, ileus within the first 6 months after hospital discharge.

Functional alterations in gut microbiota in CRC patients with ileus were analyzed using PICRUSt data to predict functional composition profiles. Of the 328 level 3 KEGG pathways evaluated, 137 pathways were significantly different between the two groups, at *Q* < 0.05 (Supplementary Table S4), and 123 differences were found between distal CRC + ileus patients and distal CRC + no-ileus patients (Wilcoxon rank-sum test, *Q* < 0.05, Supplementary Table S5). Pathways of bacterial invasion of epithelial cells, bacterial motility proteins, lipopolysaccharide biosynthesis, fatty acid metabolism, and tryptophan metabolism were clearly enriched in the ileus group compared with the no-ileus group (Figure 5A).

### Gut Microbiota Prediction of POI

Furthermore, we investigated whether the gut microbiota of preoperative CRC patients can serve as biomarkers for predicting POI in CRC patients. We divided patients into an early-onset ileus group (occurring in the postoperative perioperative period, EIG) and a delayed-onset ileus group (occurring within the first 6 months after hospital discharge, DIG) according to follow-up results. Propensity score matching based on age, sex, and CRC location was conducted, and a twofold sample size was selected as the control group for EIG and DIG. The Mann–Whitney *U* test was used to compare differences in the gut microbiota between the EIG and no-EIG groups at the genus level. Only *Faecalibacterium* showed significant differences between the two groups (*p* < 0.05). ROC curve analysis was conducted to predict the potential EIG using the abundance of *Faecalibacterium*, and we observed that the AUC of *Faecalibacterium*-based prediction was 0.74 (Figure 5B). The Youden index was employed to determine the optimal cutoff point, and -1.52 (log10 value) was selected based on the *Faecalibacterium* abundance that provided the best balance between sensitivity and specificity for predicting EIG. Overall, patients with a low abundance of *Faecalibacterium* had a high risk of POI. The prediction of *Faecalibacterium* for the DIG and no-DIG control groups was 0.67 (Figure 5B).

In addition, all CRC patients and the EIG vs. no-EIG control groups and DIG vs. no-DIG control groups were classified into high- and low-risk subsets according to the cutoff value of *Faecalibacterium* abundance derived from the EIG and no-EIG control groups. In this case, the rate of ileus in the high-risk group was significantly higher than that in the low-risk group for all CRC patients and the EIG vs. no-EIG control groups (94.12% vs. 56.86%, *p* = 0.00018, and 100% vs. 55%, *p* = 0.01123, respectively) but not for the DIG vs. no-DIG control groups (77.78% vs. 55.56, *p* = 0.25960, Figure 5D).

To further validate whether *Faecalibacterium* has a similar prediction value in POI in a different patient population, we analyzed an additional cohort of 38 patients, whereby the samples in Cohort 2 were classified into high- and low-risk subsets according to the cutoff value [-1.52 (log10 value)] of *Faecalibacterium* abundance derived from Cohort 1. The amount of *Faecalibacterium* was lower in POI patients than in non-POI patients in Cohort 2. The predictive power of the model was evaluated using validation Cohort 2, and an AUC value of 0.79 was achieved (Figure 5C). We found that the POI rate in the high-risk group was lower than that in the low-risk group (100% vs. 68.7%, *p* = 0.11067) (Figure 5E).

More interestingly, there were three common patients in both the EIG and DIG groups. After separating these three patients into a new group, we compared the difference in gut microbiota among the EIG, DIG, and new groups. With an average abundance level >0.5% at the genus level, the abundances of *unclassified Oxalobacteraceae*, *Ralstonia*, *Acinetobacter*, *Rhodococcus*, *unclassified norank*, *Burkholderia*, *Paraburkholderia*, and *Prevotella 9* were high in these three patients; conversely, *Bacteroides*, *Klebsiella*, *Fusobacterium*, *Faecalibacterium*, *Peptostreptococcus*, and *Enterobacter* were reduced (Figure 5F).

## Discussion

In the current study, we compared the gut microbiota of CRC patients with and without ileus and alterations in the gut microbiota in patients with different CRC locations with and without ileus; we further compared differences in the gut microbiota postoperatively in ileus patients. We found changes in the gut microbiota between CRC patients with and without ileus, including alpha diversity, beta diversity, and specific taxa. Similar results were found between the distal CRC + ileus and distal CRC + no-ileus groups but not the proximal groups. The F/B ratio and MDI were significantly higher in CRC patients with ileus than in CRC patients without ileus. Moreover, functions of the gut microbiota analyzed by PICRUSt differed between patients with and without ileus. Finally, we investigated the ability of the gut microbiota in preoperative CRC patients to predict POI and the difference of the gut microbiota among EIG, DIG, and overlapping patients in the two groups.

Increasing attention has been paid to the relationship between gut microbiota and the development of CRC. For example, in animal model studies, germ-free mice and colon tumor-bearing mice received feces transplants from CRC patients, revealing that the gut microbiota plays a crucial role in the development of CRC (18, 19). In clinical trials, differences of the gut microbiota between CRC patients and healthy controls have been confirmed (4–6, 20, 21), and the gut microbiota was even found to contribute to chemoresistance in CRC patients (22). However, most of these studies did not address the common complication of ileus (2), which is thought to promote microbiota overgrowth (9). Accordingly, we designed a cross-sectional study to investigate (i) microbiota differences in CRC patients with and without ileus and (ii) relationship between gut microbiota and postoperative complications (POI).

First, we analyzed gut microbial dysbiosis between patients with and without ileus. Some previous studies have shown that microbial community diversity or richness of CRC patients were significantly changed, with evenness and Shannon index values being higher in healthy controls than in CRC patients (23–25). Our results indicated no change in the Sobs index but a decrease in the Shannon index in ileus patients compared with those without ileus. These findings suggest that although CRC patients with and without ileus harbor the same taxa, abundances differ. Based on the results of reduced species richness and diversity in ileus patients, we speculate that the degree of dysbiosis in the gut microbiota is greater in CRC ileus patients than in CRC patients compared with healthy individuals. This inference was confirmed by the F/B ratio and MDI, and we observed an inverse correlation between MDI and alpha diversity. Coincident results were found for different bacterial taxa; *Escherichia–Shigella*, *Veillonella*, and *Ralstonia* were enriched in the ileus groups, whereas *Bacteroides* was depleted. *Escherichia–Shigella* was significantly more abundant in the gut microbiota of CRC patients (20), and *Veillonella dispar* has been shown to be associated with potential chemoresistance (26). However, *Bacteroides* and Bacteroidetes were depleted in CRC patients (27, 28). These results may indicate that the high rate of recurrence in CRC patients with ileus undergoing resection is related to dysbiosis of the gut microbiota.

Next, we compared differences of the gut microbiota for patients with CRC at different locations. Differences in patient demographics, clinical presentation, and tumor biology between proximal and distal colon cancers have been reported (29), and distal colon cancer is often infiltrated with obstructive symptoms (30). In our research, the rate of ileus in proximal and distal CRC did not differ significantly, consistent with previous studies (31). Furthermore, we investigated the difference in the gut microbiota for proximal and distal CRC patients with and without ileus and found a significant difference between distal CRC patients with and without ileus but not for proximal CRC patients. This result may be due to the distal CRC comparison. The microenvironment of the gut microbiota in proximal CRC is similar to that of ileus, including high bile acid levels and hypoxia, conditions that are more suitable for the survival of anaerobic bacteria (32).

We also investigated the mechanisms underlying the link between the gut microbiota and a high rate of recurrence in CRC patients with ileus. PICRUSt analysis was employed to compare the function of the gut microbiota between the two groups. In an animal experiment, Yang Y. et al., reported increased proliferation and invasive activities of bacteria. Additionally, APC^*min/+*^ mice gavaged with *Fusobacterium nucleatum* were significantly more likely to develop colorectal tumors and had a shorter survival time than mice given PBS (33). A 16S rRNA amplicon sequencing study in Morocco showed that both the gut microbiota and pathways of bacterial motility proteins and fatty acid metabolism were altered in CRC patients compared with healthy individuals (34). In our research, the above pathways were significantly increased in the ileus group compared with the no-ileus group, and the pathway of lipopolysaccharide biosynthesis was also enriched in the ileus patients. Lipopolysaccharide has been reported to upregulate VEGFR-3 expression, which promotes cell migration and invasion in CRC (35). Therefore, dysbiosis of the gut microbiota and the enriched bacteria associated with CRC may influence the recurrence of CRC in ileus patients through related pathways.

Finally, we studied the relationship between the composition of gut microbiota and gut transit time. Studies have shown that gut microbes alter the development of small bowel motility patterns, and there is a clear link between gastrointestinal motility and gut microbiota (36). Moreover, ingestion of prebiotics has been reported to accelerate transit time (37); a recent study described gut microbiota alterations in germ-free mice that received fecal microbiota from constipated patients (38). Moreover, constipation-induced changes in the gut microbiota further affect gastrointestinal motility (39).

The pathway of tryptophan metabolism was significantly higher in the ileus group than in the no-ileus group. Roager H. M. et al., found that tryptophan and 5-hydroxytryptophan were negatively associated with colon transit time. Tryptophan is the precursor to 5-hydroxytryptamine (5-HT) (40), and other researchers reported that 5-HT is negatively correlated with transit time and gut microbiota had a potential role in the pathogenesis of chronic constipation by increasing the increased expression of the 5-HT transporter (38).

As the gut microbiota and metabolites may influence transit time, we used the different bacterial taxa of preoperative CRC patients as biomarkers to predict ileus in postoperative CRC patients. The compositions of gut microbiota in the EIG and DIG patients differed from their own control groups. For the EIG and no-EIG control groups, a significant difference was found for only one bacterial taxon, and it was an independent risk factor for ileus in CRC patients. The prediction model of EIG and no EIG had a higher AUC than that of DIG and no DIG. However, this may be due to perioperative fasting, causing the gut microbiota composition in the perioperative period to be similar to that at sample collection. For DIG, different dietary habits, living environments, and radiotherapy or chemotherapy result in differences in gut microbiota (41), and it has been reported that *Faecalibacterium* and *Bacteroides* correlate positively and negatively, respectively, with colon transit in IBS (42). Our results were the same as those of Parthasarathy G. et al. (43). *Faecalibacterium* is the most abundant producer of butyrate in the gut (44), and the effects of butyrate, including decreased colon mucin secretion (45) and increased colon water and electrolyte absorption (46), may predispose individuals toward constipation. Additionally, it has been reported that butyrate can stimulate colonic motility, either directly by stimulating the release of 5-HT or indirectly by promoting cholinergic pathways (47, 48). Regardless, we did not measure the concentration of butyrate in patients’ blood or mucosa samples, and further metabolomics-based studies are necessary.

More interestingly, three patients were categorized in both the EIG and DIG groups. Because the sample size is small (three samples), we did not perform a statistical comparison, though the gut microbiota of these three patients differed. These results may serve as a basis for future research about the mechanism involved in the association between the gut microbiota and POI.

Previous studies have paid attention to the gut microbiota of CRC patients and healthy controls, whereas little attention has been paid to gut microbiota changes in CRC patients with and without ileus, even pre- and postoperation. In the present study, we explored differences in the gut microbiota and related pathways between these groups. However, there are some limitations to our study. First, 40% of the patients in our cohort had CRC, which was quite different from previous studies. In China, due to a lack of awareness of the need for a regular physical examination and especially resistance to colonoscopy, most cases of CRC are not diagnosed until complications arise. This greatly increases the proportion of ileus among patients. However, the follow-up time in this study was short, and we did not collect postoperative samples. For patients who undergo colorectal surgery, changes in intestinal physiology and anatomy, recurrence of CRC, and the development of intestinal adhesion may lead to the development of POI. Therefore, future research with longer follow-up times and multiple time-point sample collection may better illustrate the relationship between the gut microbiota and POI.

Despite these limitations, we observed different gut microbiota in CRC patients with and without ileus. Diversity analysis, the F/B ratio, and MDI revealed abnormal gut microbiota in those with ileus. The observed differences in bacterial taxa and microbiota-related pathways between CRC patients with and without ileus were in line with previous studies of CRC patients and healthy controls. More importantly, the different bacterial taxa of preoperative CRC patients might be used as biomarkers to predict the development of POI in these patients.

## Data Availability Statement

The original OTUs of Cohort 1 and Cohort 2 are shown in Supplementary Tables S6, S7.

## Ethics Statement

The studies involving human participants were reviewed and approved by the clinical trial was approved by the Research Ethics Committee of the First Affiliated Hospital of Harbin Medical University (IRB-AF/SC-08/05.0). The patients/participants provided their written informed consent to participate in this study. Written informed consent was obtained from the individual(s) for the publication of any potentially identifiable images or data included in this article.

## Author Contributions

YW and YJ made substantial contributions from the conception of the experiment to the design and acquisition of the data. YJ, RG, and YL collected the samples. YW, FZ, and JF carried out the clinical diagnosis and treatment. YL, LL, and XJ performed the bioinformatics and statistical analyses, and interpreted the data. YW, YJ, and RG wrote the manuscript. All authors read and approved the final manuscript.

## Conflict of Interest

The authors declare that the research was conducted in the absence of any commercial or financial relationships that could be construed as a potential conflict of interest.

## Abbreviations

5-HT: 5-hydroxytryptamine
AUC: area under the curve
CI: confidence interval
CRC: colorectal cancer
LDA: linear discriminant analysis
LEfSe: linear discriminant analysis effect size
MDI: microbial dysbiosis index
OTUs: operational taxonomic units
PCoA: principal coordinate analysis
PERMANOVA: permutational multivariate analysis of variance
POI: postoperative ileus
ROC: receiver operating characteristic.
