# Supplementary material for: Prediction of Postoperative Ileus in Patients With Colorectal Cancer by Preoperative Gut Microbiota
Source: Front Oncol. 2020 Nov 25;10:526009. doi: 10.3389/fonc.2020.526009 (PMC7724052; doi:10.3389/fonc.2020.526009)

Prediction of postoperative ileus in patients with colorectal cancer by preoperative gut microbiota

Ye Jin[1, 2, 3 *], Rui Geng[1,4 *], Yang Liu[1, 3], Lujia Liu[1], Xiangren Jin[1], Lei Zhao[1], Fuya Zhao[1], Jing Feng[1], Jiayu Sun[1], Baiqiang Lin[1], Yunwei Wei[1, 3 +]

[1]Department of Oncological and Laparoscopic Surgery, the First Affiliated Hospital of Harbin Medical University, Harbin, China, 150001

[2]Department of Hepatic Surgery, the First Affiliated Hospital of Harbin Medical University, Harbin, China, 150001

[3]Key Laboratory of Hepatosplenic Surgery, Ministry of Education, The First Affiliated Hospital of Harbin Medical University, Harbin, China, 150001

[4]Department of Thyroid and Breast Surgery, the First Affiliated Hospital of University of Science and Technology of China, Hefei, China, 230000

[+]Corresponding Author: Yunwei Wei, No. 23 Youzheng Street, Nangang District, Harbin, China; phone: +86-0451-85555729; hydwyw11@hotmail.com.

[*]These authors contributed equally to this work.

Figure S1. The relative abundance of faecal bacterial family and genus were clustered into ileus and no ileus patients. All OTUs with lower abundances were grouped as "others".

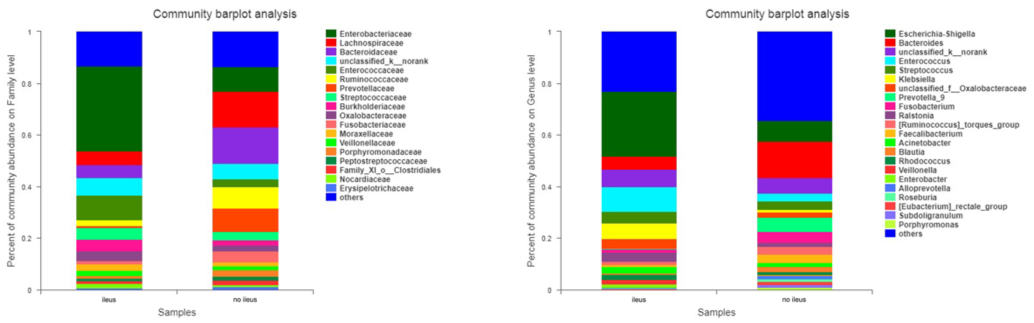

Figure S2. The (A) Sobs, (B) Shannon and (C) Simpson index on OTU of patients with distal ileus and proximal ileus. (D) The microbial dysbiosis index of ileus and no ileus patients at the genus level. (E) Principal coordinate analysis of the weighted UniFrac distance between distal ileus and proximal ileus group. ***p<0.001

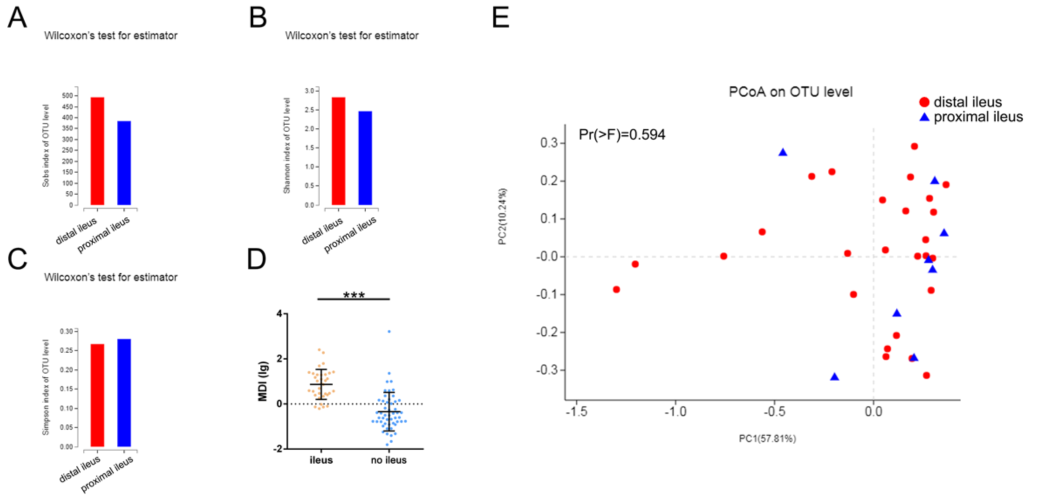

Supplement: Supplementary file 1 [file Image_1.PDF]
