# Supplementary material for: Prediction of Postoperative Ileus in Patients With Colorectal Cancer by Preoperative Gut Microbiota
Source: Front Oncol. 2020 Nov 25;10:526009. doi: 10.3389/fonc.2020.526009 (PMC7724052; doi:10.3389/fonc.2020.526009)
Supplement: Supplementary file 3 [file Data_Sheet_2.DOCX]

| **Species name** | **Mean** | **LDA_value** | **Pvalue** |
| --- | --- | --- | --- |
| **Phylum** |  |  |  |
| *Proteobacteria* | 5.78 | 5.31 | 4.01E-06 |
| *Bacteroidetes* | 5.47 | 5.13 | 5.45E-08 |
| *Firmicutes* | 5.51 | 4.80 | 2.43E-02 |
| *Fusobacteria* | 4.80 | 4.61 | 1.41E-04 |
| **Family** |  |  |  |
| *Enterobacteriaceae* | 5.62 | 5.15 | 4.83E-05 |
| *Bacteroidaceae* | 5.31 | 4.91 | 7.76E-06 |
| *Lachnospiraceae* | 5.09 | 4.71 | 4.76E-06 |
| *Prevotellaceae* | 4.94 | 4.60 | 2.25E-03 |
| *Fusobacteriaceae* | 4.80 | 4.42 | 1.41E-04 |
| *Ruminococcaceae* | 4.72 | 4.39 | 7.11E-07 |
| *Burkholderiaceae* | 4.86 | 4.37 | 3.91E-02 |
| *Veillonellaceae* | 4.29 | 4.20 | 2.58E-02 |
| *Family XI o Clostridiales* | 4.19 | 4.18 | 1.85E-03 |
| *Family XI o Bacillales* | 4.09 | 4.17 | 1.78E-02 |
| *Coriobacteriaceae* | 4.16 | 4.00 | 1.90E-02 |
| *Peptostreptococcaceae* | 4.20 | 3.99 | 4.09E-03 |
| **Genus** |  |  |  |
| *Escherichia_Shigella* | 5.52 | 5.03 | 1.65E-04 |
| *Bacteroides* | 5.31 | 4.92 | 7.76E-06 |
| *Prevotella_9* | 4.94 | 4.61 | 2.25E-03 |
| *Faecalibacterium* | 4.72 | 4.42 | 7.11E-07 |
| *Fusobacterium* | 4.80 | 4.41 | 1.41E-04 |
| *Ruminococcus torques group* | 4.75 | 4.39 | 9.34E-06 |
| *Ralstonia* | 4.75 | 4.31 | 1.20E-02 |
| *Eubacterium rectale group* | 4.46 | 4.23 | 7.58E-04 |
| *Gemella* | 4.09 | 4.18 | 1.78E-02 |
| *Veillonella* | 4.29 | 4.17 | 2.58E-02 |
| *Parvimonas* | 4.19 | 4.17 | 1.85E-03 |
| *Blautia* | 4.32 | 4.13 | 6.80E-05 |
| *Roseburia* | 4.25 | 4.10 | 6.58E-05 |
| *Collinsella* | 4.16 | 3.99 | 1.90E-02 |
| *Peptostreptococcus* | 4.20 | 3.99 | 4.09E-03 |

**Table S2. The LEfSe analysis of gut microbiota between ileus and no ileus CRC patients.**

LDA, linear discriminant analysis.
