# Supplementary material for: Prediction of Postoperative Ileus in Patients With Colorectal Cancer by Preoperative Gut Microbiota
Source: Front Oncol. 2020 Nov 25;10:526009. doi: 10.3389/fonc.2020.526009 (PMC7724052; doi:10.3389/fonc.2020.526009)
Supplement: Supplementary file 4 [file Data_Sheet_3.DOCX]

| **Species name** | **ileus** | **no ileus** | **p_value** | **Q value** |
| --- | --- | --- | --- | --- |
| **Phylum** |  |  |  |  |
| *Proteobacteria* | 60.9±31.1 | 26.8±28.9 | 4.10E-06 | 1.23E-05 |
| *Firmicutes* | 24.6±30.3 | 32.5±22.0 | 2.46E-02 | 3.70E-02 |
| *Bacteroidetes* | 5.8±10.0 | 29.3±23.5 | 5.58E-08 | 3.35E-07 |
| *Fusobacteria* | 1.9±6.9 | 6.3±13.5 | 1.43E-04 | 2.87E-04 |
| **Family** |  |  |  |  |
| *Enterobacteriaceae* | 41.6±33.7 | 14.2±22.7 | 4.93E-05 | 2.34E-04 |
| *Bacteroidaceae* | 5.7±10.0 | 20.5±19.9 | 7.92E-06 | 5.02E-05 |
| *Lachnospiraceae* | 2.8±5.2 | 12.4±10.8 | 4.87E-06 | 4.62E-05 |
| *Burkholderiaceae* | 7.2±12.3 | 3.7±7.8 | 3.95E-02 | 6.26E-02 |
| *Prevotellaceae* | 0.1±0.4 | 8.8±18.2 | 2.29E-03 | 6.21E-03 |
| *Fusobacteriaceae* | 1.9±6.9 | 6.3±13.5 | 1.43E-04 | 5.45E-04 |
| *Ruminococcaceae* | 0.8±2.1 | 5.2±5.8 | 7.28E-07 | 1.38E-05 |
| *Peptostreptococcaceae* | 0.8±2.5 | 1.6±2.9 | 4.15E-03 | 9.85E-03 |
| *Veillonellaceae* | 2.0±10.4 | 0.4±1.0 | 2.61E-02 | 4.50E-02 |
| *Family Bacillales* | 0.7±2.1 | 1.2±3.6 | 1.80E-02 | 3.66E-02 |
| *Coriobacteriaceae* | 0.5±1.1 | 1.4±2.4 | 1.92E-02 | 3.66E-02 |
| *Family Clostridiales* | 0.3±1.0 | 1.6±4.2 | 1.88E-03 | 5.95E-03 |
| **Genus** |  |  |  |  |
| *Escherichia Shigella* | 32.8±30.5 | 12.3±22.2 | 1.68E-04 | 5.99E-04 |
| *Bacteroides* | 5.7±10.0 | 20.5±19.9 | 7.92E-06 | 7.95E-05 |
| *Prevotella 9* | 0.1±0.4 | 8.8±18.2 | 2.29E-03 | 5.72E-03 |
| *Ralstonia* | 5.7±11.3 | 2.9±7.2 | 1.22E-02 | 2.53E-02 |
| *Fusobacterium* | 1.9±6.9 | 6.3±13.5 | 1.43E-04 | 5.97E-04 |
| *Ruminococcus torques group* | 1.6±3.9 | 5.7±7.0 | 9.54E-06 | 7.95E-05 |
| *Faecalibacterium* | 0.8±2.1 | 5.2±5.8 | 7.28E-07 | 1.82E-05 |
| *Eubacterium rectale group* | 0.2±0.4 | 2.9±5.2 | 7.71E-04 | 2.41E-03 |
| *Blautia* | 0.6±1.1 | 2.1±2.5 | 6.93E-05 | 3.46E-04 |
| *Peptostreptococcus* | 0.8±2.5 | 1.6±2.9 | 4.15E-03 | 9.42E-03 |
| *Veillonella* | 2.0±10.4 | 0.4±1.0 | 2.61E-02 | 4.35E-02 |
| *Roseburia* | 0.4±1.2 | 1.8±3.9 | 6.71E-05 | 3.46E-04 |
| *Gemella* | 0.7±2.1 | 1.2±3.6 | 1.80E-02 | 3.44E-02 |
| *Collinsella* | 0.5±1.1 | 1.4±2.4 | 1.92E-02 | 3.44E-02 |
| *Parvimonas* | 0.3±1.0 | 1.6±4.2 | 1.88E-03 | 5.22E-03 |

Table S3. The Mann–Whitney U test of gut microbiota between ileus and no ileus CRC patients with Q<0.05.
